# Supplementary figures and images for: Prevention of the Pro-Aggressive Effects of Ethanol-Intoxicated Mice by Schisandrin B
Source: Nutrients. 2023 Apr 15;15(8):1909. doi: 10.3390/nu15081909 (PMC10146817; doi:10.3390/nu15081909)

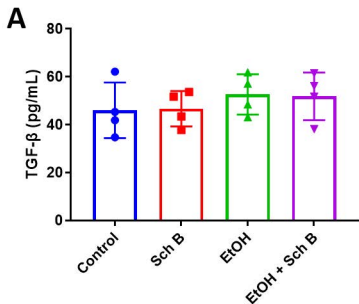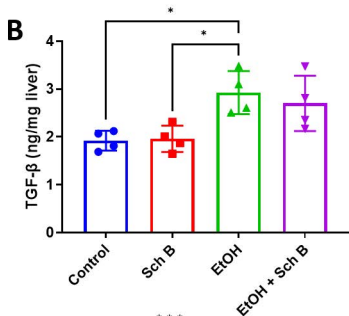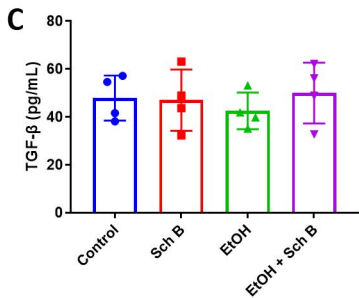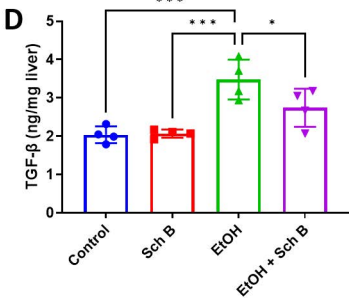

Supplement: Supplementary file 1 [file nutrients-15-01909-s001.zip › nutrients-2297245-supplementary.pdf]
